# “Language Breathes Life”—Barngarla Community Perspectives on the Wellbeing Impacts of Reclaiming a Dormant Australian Aboriginal Language

**DOI:** 10.3390/ijerph16203918

**Published:** 2019-10-15

**Authors:** Leda Sivak, Seth Westhead, Emmalene Richards, Stephen Atkinson, Jenna Richards, Harold Dare, Ghil’ad Zuckermann, Graham Gee, Michael Wright, Alan Rosen, Michael Walsh, Ngiare Brown, Alex Brown

**Affiliations:** 1 South Australian Health and Medical Research Institute—PO Box 11060, Adelaide, South Australia 5000, Australia; seth.westhead@sahmri.com (S.W.); ngiareb@gmail.com (N.B.); Alex.Brown@sahmri.com (A.B.); 2 University of Adelaide, Adelaide, South Australia 5005, Australia; Emma.Richards@adelaide.edu.au (E.R.); Ghilad.Zuckermann@adelaide.edu.au (G.Z.); 3 Barngarla Language Advisory Committee (South Australian Health and Medical Research Institute)—PO Box 11060, Adelaide, South Australia 5000, Australia; statkinson72@gmail.com (S.A.); jenna21richards@hotmail.com (J.R.); harold.dare@gmail.com (H.D.); 4 Murdoch Children’s Research Institute—Flemington Road, Parkville, Victoria 3052, Australia; Graham.Gee@mcri.edu.au; 5 University of Melbourne, Melbourne, Victoria 3010, Australia; 6 Curtin University—Kent Street, Bentley, Western Australia 6102, Australia; M.Wright@curtin.edu.au; 7 University of Sydney, Sydney, New South Wales 2006, Australia; alanrosen@med.usyd.edu.au (A.R.); Michael.Walsh@sydney.edu.au (M.W.); 8 University of Wollongong—Northfields Ave, Wollongong, New South Wales 2522, Australia; 9 Australian National University, Canberra, Australian Capital Territory 0200, Australia

**Keywords:** Australia, Aboriginal and Torres Strait Islander health, social and emotional wellbeing, Indigenous language, Revivalistics

## Abstract

Traditional languages are a key element of Indigenous peoples’ identity, cultural expression, autonomy, spiritual and intellectual sovereignty, and wellbeing. While the links between Indigenous language loss and poor mental health have been demonstrated in several settings, little research has sought to identify the potential psychological benefits that may derive from language reclamation. The revival of the Barngarla language on the Eyre Peninsula, South Australia, offers a unique opportunity to examine whether improvements in mental health and social and emotional wellbeing can occur during and following the language reclamation process. This paper presents findings from 16 semi-structured interviews conducted with Barngarla community members describing their own experienced or observed mental health and wellbeing impacts of language reclamation activities. Aligning with a social and emotional wellbeing framework from an Aboriginal and Torres Strait Islander perspective, key themes included connection to spirituality and ancestors; connection to Country; connection to culture; connection to community; connection to family and kinship; connection to mind and emotions; and impacts upon identity and cultural pride at an individual level. These themes will form the foundation of assessment of the impacts of language reclamation in future stages of the project.

## 1. Introduction

For Indigenous peoples, the protection, preservation and celebration of culture is a central tenet and core demonstration of their sovereign rights [1]. Article 13 of the United Nations Declaration on the Rights of Indigenous Peoples [2] states that:

“Indigenous peoples have the right to revitalize, use, develop and transmit to future generations their histories, languages, oral traditions, philosophies, writing systems and literatures…”

Traditional languages are a key element of Indigenous peoples’ identity, cultural expression, autonomy, and spiritual and intellectual sovereignty. As such, they are vital to wellbeing [3]. However, for many Indigenous peoples globally, the processes of colonisation have involved the systematic separation of people from their languages. Linguicide (language killing) and glottophagy (language eating) have been in operation in Australia since the early colonial period, when efforts were made to prevent Aboriginal and Torres Strait Islander people from continuing to speak their languages in order to “civilize” them [4]. Even former Governor of South Australia (1841–1845), George Grey, remarked in his journal that “the ruder languages disappear successively, and the tongue of England alone is heard around” (Grey 1841: 200-01, in [4]). What was seen as a civilising process was actually the traumatic death of many Aboriginal and Torres Strait Islander languages, and the associated destruction of intellectually sophisticated cultural beliefs, practices, and activities that had been transferred from one generation to the next for more than 50,000 years. Blatant statements of linguistic imperialism, such as the ones made by Grey, now seem to be less frequent, but the processes they describe are nonetheless still active. Thus, the decline of Indigenous languages has become a prevalent characteristic of colonisation, negatively impacting the wellbeing of Indigenous populations over time [5]. Pervasive racism, marginalisation, family disconnectedness, community dysfunction, and social disadvantage constitute chronic causes of stress among Aboriginal communities and have ongoing effects on the mental health and wellbeing of individuals [6,7]. In particular, it is the loss of land, culture, and identity that have been highlighted by Indigenous people as fundamental causes of ill health [8]. 

The link between language loss and poor mental health has been demonstrated in a number of settings. For example, Hallett and colleagues [9] have shown a strong correlation between youth suicide and lack of conversational knowledge in native languages in Canada. In Australia, language loss has been shown to have negative impacts, with high levels of acculturative stress seen in children living in regional centres where language loss was occurring [10]. Language loss is now a worldwide phenomenon, disproportionately affecting Indigenous peoples, with expectations that at least half of the world’s languages will be lost this century [3,11]. In Australia, only 13 Aboriginal languages are now widely spoken [3], constituting only 4% of the approximately 330 pre-contact languages. Since 1991, the proportion of Aboriginal and Torres Strait Islander people who reported speaking an Australian Indigenous language at home has decreased from 16 per cent to 10 per cent. In the 2016 Census, 63,754 people reported speaking an Australian Indigenous language at home [12]. Australian Geographic reported a similar set of trends, noting that of the 250 distinct Indigenous languages that were spoken at the time of European colonisation of Australia, 110 now are considered severely or critically endangered [13]. 

However, the Second National Indigenous Languages Survey [3] identified an overwhelming desire among Australian Indigenous communities to reclaim their traditional languages because of the potential of language reclamation to impact on cultural renewal, cultural identity and wellbeing. Furthermore, it found that the majority of contemporary language reclamation activities within Australia were not perceived by Aboriginal people as being aimed, linguistically, at increasing the number of language speakers, but rather as helping people to reconnect with their culture, build and maintain cultural identity, and improve wellbeing. Language reclamation was seen to provide benefits through strengthening identity and a sense of belonging; empowering Indigenous people by increasing self-esteem and a sense of pride; and improving communication between communities, families and across generations [3]. 

Nevertheless, despite some promising indications [9,14,15], little research has sought to identify the potential psychological benefits that may derive specifically from language reclamation, and to date there has been no systematic study of the impact of language revival on mental health and wellbeing. 

The Barngarla people of the Eyre Peninsula in South Australia are one example of Aboriginal people suffering the effects of linguicide. In response, Barngarla community members in Port Augusta and Port Lincoln have been working with linguists and language revivalists since 2012 to reclaim, re-learn, document, and transmit their language to the next generation [16]. The revival of the Barngarla language offers a unique opportunity to examine whether improvements in mental health and social and emotional wellbeing can occur during and following the language reclamation process. The Barngarla Language and Wellbeing Study is a five-year National Health and Medical Research Council funded project that aims to systematically assess the mental health and social and emotional wellbeing impacts of language reclamation within Barngarla communities in Port Lincoln and Port Augusta [17]. The study hypothesis is that there will be significant improvements in mental health and social and emotional wellbeing during and following the language reclamation process. 

The project has four main objectives: (I) to further develop, deliver and evaluate language reclamation activities; (II) to better understand the positive impacts of the existing, pilot language reclamation activities through semi-structured interviews with participants; (III) to review, adapt and/or develop quantitative methods for assessing mental health and social and emotional wellbeing in relation to language reclamation; and (IV) to assess language use and social and emotional wellbeing of community-based language reclamation participants in comparison with other Aboriginal members of the broader Eyre Peninsula population.

This paper presents the findings from Objective II: qualitatively exploring the perceived impacts of language activities from the perspective of Barngarla people. The “domains of impact” that were identified by community members will be used to guide the subsequent development of an assessment tool for measuring the impacts of language reclamation (Objective III). As well as being used for the remainder of this study, it is hoped that the resulting assessment tool may have broader applicability for measuring the impacts of Indigenous language reclamation activities both within Australia and internationally. 

## 2. Materials and Methods 

### 2.1. Decolonising Methodologies

This study applies an Indigenous research paradigm [18,19,20,21,22,23,24,25], which prioritises the “lifeworlds” [26] and voices of Indigenous people; whether literally, as illustrated in our results section, or through our selection of published supporting evidence. This is in order to demonstrate our recognition of Aboriginal and Torres Strait Islander peoples’ intellectual sovereignty, and to redress inaccurate portrayals of Aboriginal and Torres Strait Islander people’s intellectual sophistication that had characterised earlier colonial-influenced research in Australia. A recently published definition of Indigenous methodologies is as follows:

*“Indigenous methodologies make visible within the research process what is meaningful and logical in Indigenous understandings of ourselves and the world [27]. An Indigenous methodology, therefore, is a methodology where the approach to, and undertaking of, research process and practices take Indigenous worldviews, perspectives, values and lived experience as their central axis” [26]*.

The considerable benefits of Indigenous people’s participation in research within their own communities has been documented by Kelly and colleagues [28], who note that, “Most important for the community researchers on this project was the sense that they were doing important health work, not just conducting research. […] Whilst research outcomes are undoubtedly important, in many cases the process used is of greater importance” (p.1).

### 2.2. Aboriginal Governance and Leadership

The project is overseen by the Barngarla Language Advisory Committee (BLAC), who, in partnership with researchers from the University of Adelaide and the Wardliparingga Aboriginal Health Equity research team in the South Australian Health and Medical Research Institute, are leading this work as they seek to reclaim their traditional language. BLAC provides advice and direction to ensure that culturally sensitive and respectful research processes guide the development of project materials and language resources for communities and the linguistic community. BLAC also represents community interests within the research team, particularly as they relate to principles of self-determination, informed consent, impacts, and benefits.

In addition to BLAC members, the remainder of the research team draws heavily on Aboriginal expertise from a range of perspectives. Of the 14 study team members, three of the four Chief Investigators, three of the five Associate Investigators and two further members of BLAC are Aboriginal, as is the Research Assistant. Non-Indigenous team members include two linguists, one community psychiatrist, a senior research fellow, and the study coordinator. 

### 2.3. Ethics

This project was approved by the Aboriginal Health Research Ethics Committee of South Australia (AHREC 04-17-708; 04-18-768) and the University of Adelaide Human Research Ethics Committee (H-2017-085).

### 2.4. Study Design 

We follow the consolidated criteria for reporting qualitative research (COREQ) as proposed by Tong and colleagues [29]. Given the innovative nature of this project, incorporating a developmental phase that centres on the experiences of Aboriginal communities is critical to engaging local community members in the conduct of the research. As well as more clearly identifying the outcomes of interest to the communities, the completion of Objective II provides a historical record of the impacts of the initial reclamation workshops and contributes to the further development of the evaluation framework for the project from the perspective of community members themselves. 

While the overall study employs a mixed methods approach framed within a longitudinal cohort study, the developmental phase began with qualitative methods that align with the principles of Participatory Action Research, including deliberately sharing power and advocating for the active involvement of community members in undertaking the research itself [30,31]. At the outset of the developmental phase, a two-day workshop was convened with BLAC to begin discussing and documenting the domains of interest that would form the basis of interviews with pilot workshop participants and their families. This process replicates a similar approach to that undertaken in developing qualitative health services research methods in Aboriginal communities [32,33,34,35]. 

### 2.5. Community Engagement and Development of Research Questions

Prior to undertaking fieldwork in late October and early November 2017, the project team began engaging with Barngarla community members and introducing the proposed study in Port Augusta and Port Lincoln in April 2017, with subsequent visits by the Study Coordinator and/or Research Assistant in August and October 2017. In late May 2017, members of BLAC had taken part in the two-day Investigator Workshop to outline the study and begin discussing possible interview questions for the qualitative fieldwork. Following the workshop, draft questions were circulated to BLAC members and Investigators for review and comment before being refined. 

The semi-structured interview questions focused on participants’ experiences to ascertain their perceptions of the health and wellbeing impacts of language revival activities. The questions took a broad definition of health and wellbeing that provided prompts around physical, mental, emotional, social and cultural aspects of health, but was fundamentally left open to the participants to determine and define in their own ways. The interview schedule began with demographic context, followed by questions regarding participants’ experiences with Aboriginal languages generally, and Barngarla language specifically, before prompting around potential health impacts. The interviews also explored what key language-related activities were believed to support improvements in individual, family, or community wellbeing. 

### 2.6. Procedures

A convenience sample of participants was recruited by the Community Research Associates (ER and SA), who also provided transport to and from the interviews for participants, as required. While most participants were interviewed at central locations, one group chose to be interviewed at home in order to accommodate the care of pre-school aged children. Interviews were catered with food or drink, but no other participant payment was made. 

The interviews were conducted by the Study Coordinator (LS) and Research Assistant (SW), both of whom are based at Wardliparingga and experienced in qualitative research. Participants were offered the opportunity to be interviewed one-on-one or in groups clustered by age or gender, as the Research Assistant is a younger, Aboriginal male, while the Study Coordinator is an older, non-Indigenous female. However, almost all interviews were conducted by both interviewers together, while alternating the role of lead interviewer. This allowed the interviewers to discuss and reflect on each conversation as the fieldwork progressed, to share differing perspectives, identify potential assumptions, triangulate interpretations, and thereby to mediate observer bias.

Following the receipt of informed consent, participants were asked to discuss their experiences and perceptions regarding Barngarla language revival activities. Interviews lasted between 40–90 min and most were digitally audio recorded. Detailed fieldnotes were taken when participants chose not to be audio recorded. Fieldnotes also were used to assist with transcribing and to take note of contextual details and non-verbal expressions. Audio recordings of the interviews were transcribed by an independent professional transcription service, then listened to again by the interviewers while they de-identified the transcripts in order to ensure accuracy and to re-familiarise the interviewers with each conversation. The de-identified transcripts (or notes from non-recorded interviews) were then sent back to participants for their review and revision (i.e., member checking) prior to being analysed. 

### 2.7. Analysis and Interpretation

Analysis was thematic, involving a deductive approach using the electronic software program NVivo to identify analytic categories directly from the interview material. The two researchers who conducted the interviews (LS and SW) undertook independent thematic analyses of the transcripts, then discussed the coding in order to identify and resolve differences in interpretation. Themes were checked back against the transcriptions to ensure consistency and validity.

### 2.8. Feedback and Consensus

Feedback of the preliminary findings to community members took place in Port Lincoln on 20 December 2017 and Port Augusta on 24 January 2018. The purpose of the feedback sessions was to seek community consensus as to whether emerging themes had been interpreted correctly; to provide an opportunity to identify any other impacts on health and wellbeing that were important for the study but that did not come through in the initial interviews; and to request community-wide permission to use the findings to progress the agreed study objectives. Overall, feedback from both Port Augusta and Port Lincoln reinforced findings and, with minor adjustments, approved the way the findings were organised and presented. Following this community consensus building process, the results were presented to the full investigator team on 2 March 2018.

## 3. Results

### 3.1. Participant Characteristics

Interviews were conducted with a total of sixteen Barngarla community members, six in Port Lincoln and ten in Port Augusta, in late October and early November 2017. Of these sixteen participants, ten were female and six were male; six were between the ages of 15–29, seven were aged 30–50, and three were over the age of 50. These latter three were all members of the Stolen Generations, who had been forcibly removed from their families and communities by the government as children, only able to return in adulthood. Given the small sample size, and in order to ensure the anonymity of all participants, quotes will not be identified by age, gender, nor location of interview.

### 3.2. Themes 

Within the context of the broader study, the main purpose of Objective II of the Barngarla Language and Wellbeing Study was to use the direct experiences and perceptions of Barngarla people to understand and document the positive impacts of language reclamation activities in order to inform the development of a quantitative tool to assess the impacts of Indigenous language reclamation in future stages of the project. The translation of these themes into domains of impact, as well as a detailed description of the development of the psychometric assessment tool, will be presented elsewhere (manuscript forthcoming).

**Figure 1:**
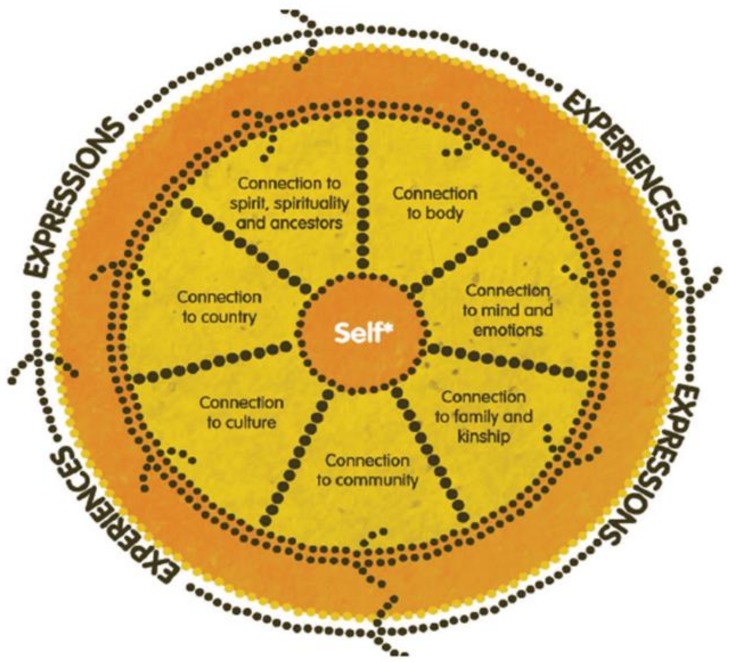
Social and Emotional Wellbeing from an Aboriginal and Torres Strait Islanders’ Perspective.

This paper explores the qualitative findings more comprehensively and organises the main themes according to a framework for “Social and Emotional Wellbeing from an Aboriginal and Torres Strait Islanders’ Perspective” [36], which is presented in Figure 1 below. This is because the themes that emerged through the initial deductive analysis subsequently aligned closely with seven of the eight domains of wellbeing presented within this framework. The relevant domains—which include connection to spirit, spirituality, and ancestry; connection to Country; connection to culture; connection to family and kinship; connection to community; connection to mind and emotions; and self—form headings for the presentation of the findings. This conception of self is grounded within a collectivist perspective that views the self as inseparable from, and embedded within, family and community.

#### 3.2.1. Connection to Spirit, Spirituality and Ancestors

Participants spoke of language and wellbeing in relation to connections to spirit and spirituality, with one participant describing language in terms of breath.

“Language breathes life. Like we talked about breathing life back into the land, and it’s that ancient language.”

This connection with spirituality was also seen as important to healing by reconnecting people with culture, heritage and the ancient spirits of the land and of the people who had gone before them.

“And it’s that spirit of the land, of their heritage, their culture, their people that are going, ‘We’re not going to let you forget who you are and where you’re from.’ And when you’re having a moment of, despair or depression or—you know, I’ve seen people go right down to the bottom. […] And it’s been their heritage, and culture, and their identity bring them back. Bring them back and ground them to who they are and where they’re from.”

People also emphasised the importance of reviving Barngarla language as a way of honouring Elders and ancestors. As one participant commented, “It’s about going into the future but also honouring the people that have passed and what they would have wanted.” 

This honouring of ancestors and Elders frequently was expressed in relation to the generations who had been unable to retain their language due to assimilation policies, including the forcible removal of children, and for those who had passed on.

“If they were alive today, people that have passed away, they would have loved to be here. And that’s where, sometimes I’ll drive along, and I’ll smile to myself about words [a departed Elder] always used to say—and then when I’m going and doing something, I smile because it’s like, ‘We’re doing it now’, you know what I mean? And he’s looking down. This is what he wanted, everything to be Barngarla and Barngarla to be recognised. So even though they’re not here, it’s about honouring their legacy that they started and that they wanted to finish.”

#### 3.2.2. Connection to Country

Connection to Country was another way in which people reflected upon the impacts of language reclamation. As one participant expressed, “The land and language are part and parcel—one and the same.” Other participants also spoke of how language revival reinforces their connection to Country, noting that land and language are intertwined.

“I think [Barngarla language] has been the final thing to bring back spirit into land. Because people went, and they connected, and wanted to learn that ceremony, and wanted to learn them stories and all. But it all comes back to song. It all comes back to words. It comes back to putting that spirit back into Country through song.”

This same participant noted the importance of speaking Barngarla language outside, on Barngarla Country rather than just in classroom workshops, again in terms of spirit and breath.

“When we are talking inside, it’s just us talking. When you’re talking outside, you’re breathing language into the land and into the sea and into the air and into the birds and into the fish and into the trees and you’re awakening that with all that spirit. You’re speaking life into all our ancient spirits out there and they’re sitting around listening.”

#### 3.2.3. Connection to Culture

Participants described the impacts of language reclamation in terms of strengthening their connection to culture, noting the interconnectedness of language, culture, Dreaming, land and sea, and other living beings. “Dreaming” refers to Aboriginal understandings of the world, of its creation, and its stories. The Dreaming is the beginning of knowledge, from which came the laws of existence. The Dreaming also indicates “a psychic state in which or during which contact is made with the ancestral spirits, or the Law, or that special period of the beginning” [37].

“I think it’s very important that we, as Barngarla people, get to learn what’s *us*, what makes us, *us.* And that’s our language and that’s our culture and that’s our Dreaming. Those three there are interconnected with who we are as Aboriginal people.”

They also described the power of connections to culture and heritage and to the strength of past generations, particularly in relation to health and wellbeing.

“So, I think your heritage and culture, and your identity, is something that—especially nowadays when people struggle with their health and wellbeing—the power of who you are and where you come from, and just the resilience that your people went through in maintaining their heritage, their culture, just in fighting to still be alive today is so powerful. So there’s a lot of strength that comes through so many generations.”

Several participants spoke of language as having been the missing part of their larger connection to culture, describing this as building toward a sense of wholeness, as the missing piece of a jigsaw puzzle, as the other half of a person’s self.

“I had my sisters and brothers around me but there was something missing. And that was being able to communicate properly with them. On face-to-face language, and I didn’t have a language. All I had was English.”

“Being an Aboriginal person, culture is the main thing. […] If you can’t speak your own language, there’s that piece missing. And when you can speak your own language, learn it there, you live in your culture, you’re speaking your culture’s language, everything learnt there, all come together, it’s the missing part of the jigsaw puzzle. […] You are getting that empowerment to speak your own language. It feels good, like lifting a burden off your side, like something is missing from a jigsaw puzzle. It’s that little puzzle piece missing, and it’s finally getting put back there, feel complete then.”

“With learning my language, it’s like I’ve learned another part of me. I can tell you now that before I learned that I had a language, I knew that I was Aboriginal, but I didn’t know which tribe or anything. And once I learned that I was Barngarla, and my language, it was like my other half came in. I don’t really know how to explain it. It was like a sense of who you are, a sense of wellbeing.”

Language reclamation was also described as facilitating the ongoing transfer of cultural knowledge. At times, this theme was grounded in the experiences of forced removal and the impacts of Stolen Generations upon Barngarla families.

“With all my mother, my uncles and aunties all being taken away, they missed out on all of those songs. That was their birthright. That was something they should have [had] as young children growing up with their grandparents or whoever. And they missed out. So, to be able to collect that and be able to give that back to the next generation, I think is—it’s awesome.”

The necessity of transferring culture was described as a legacy and an obligation, as one participant commented:

“It’s an obligation to me, it’s an obligation to every Barngarla person to make sure that we [reclaim] what we didn’t have.”

In illustrating this, several participants spoke of the importance of continuing to transfer cultural knowledge in terms of learning Barngarla language in order to teach children, grandchildren and future generations.

“The reason why we want keep on doing it is because we want our children to learn, our grandchildren to learn. I want to learn myself because that’s my language and I feel like I’ve got to learn it first before my kids and my grandkids, you know what I mean? But, I’m really happy that we’re all doing it together as a family. That’s the best thing about it, that I’m doing it with my children, I’m sharing it with my children, and my grandchildren.”

“Or we could be going down the beach and she’ll say, ‘What’s the Barngarla word for this, Mum, and what’s the Barngarla for that?’ So I need to learn it because they [young kids] are just thirsty for it. They want it, you know. […] And they really come out with things that make you go, ‘Wow! They are paying attention!’ And they are going to be so strong in it. And you don’t realise but, from that young age, that’s where it does all come along, and filters down. And they just gain their strength from it.”

“Everyone knows nursery rhymes and things from when they were kids and you never forget it. If we had Barngarla songs like that, that we could sing to our kids, our grandkids when they’re going to bed or whatever, you know, that will be imprinted into their brain and that will be forever. […] So, you’re singing these to the children every day, every night and it’s just going to be imprinted, it’s going to be there, and they’ll never get rid of it. They’ll sing that to their children and they’ll sing it to their children. And it’ll just go on forever hopefully.”

#### 3.2.4. Connection to Family and Kinship

Participants described how language activities supported the strengthening of connections to family and kinship, at times within the context of the Stolen Generations.

“I think the thing that sort of helped the family come together and start reconnecting with language, heritage and culture was really powerful. Because it started to show them, regardless of what’s happened [when they were removed], ‘You come from this long line of strong people. And your spirit is resilient. You came home. You came home to family. Your next generation—you need to be able to talk through what you went through, so they can understand.’”

Others noted how language activities have provided opportunities to spend time with family members and bring several generations together, as well as the importance of sharing both memories from the past and hopes for the future and for future generations.

“It’s also enjoyable and you get to speak to other family members who you don’t normally see. Usually the only time us blackfellas get together is like funerals, you know. So, it’s another way of us getting together and being able to enjoy what we have, and that’s the Barngarla language.”

“Interacting with the family members there, as they help each other with the painting styles, and listen to each other’s stories, tell each other stories. So, it’s good to see. And not only that, the younger generation is involved in it too. So, you have the grandmothers, you have the mothers, you have the sons, niece, grandchildren, all mixing, and you don’t sort of see that that much these days, you know, and it’s good to actually see it.”

Several participants talked about how feelings of belonging also improved as a result of language activities. This included reconnecting and forming closer bonds with family members and the extended Barngarla community, as well as working toward shared goals and achieving change together.

“The languages have an impact on that as well because there’s this continual gathering. We’re starting to gather now, and we’re not sort of scattered. I’d say that has had an impact.”

“It brings me back and into that family circle and I can walk along with them now and not sort of on that outside. So, it brings me back in and it gives me a sense of belonging again, if you can say that, and looking forward to something that’s going to help the family, going to help the group, the tribal group and the generations to come. […] It brings a sense of belonging again and community and being able to achieve something together instead of separately. It’s bringing us together.”

“The language and the language programs have brought us together. As well, I guess being in my land and surrounded by my family and learning my language has made me a lot happier.”

#### 3.2.5. Connection to Community

Participants described how feelings of connection to community were enhanced by being involved in language reclamation. They noted the value of bridging gaps and closing divides between Aboriginal people and the non-Indigenous community, as well as influencing change within their local communities, both of which often required working in “two worlds”.

“I felt what I was doing was just out there sharing my heritage and culture, as well as bridging the gap between Aboriginal [people] and the wider community. […] I was just bridging the gap between everyone.”

“So, working in the wider community, you have to work in both worlds. You can’t exclude yourself and just be all about Aboriginal people. Because we live in a society where we have to bring it forward. It’s the only way that we’re going to make it work and sustain it. […] And it’s good. Because it shows that we have influenced change here. These are people that want to acknowledge that they are on your traditional lands, and they want to meet you. Because they see you around, they see your kids around. […] And I believe the wider community’s interested in that as well, you know, the language and what we call things.”

The importance of language for healing Barngarla community and spirit was also noted, particularly in the context of experiences of previous exclusion.

“Things have shifted, people’s negativity has shifted. There is a lot of good spirit work happening here, and a lot of people feeling that now. […] They look at us and how happy we are just doing our heritage, our culture and our language.”

Participants also described Barngarla language teaching as a reconciliatory tool especially insofar as non-Indigenous children and their families can begin engaging with Aboriginal languages and cultures. Also, Barngarla children and young people have been able to engage and network with people who are or will become power brokers within the broader community.

“We’ve always seen Barngarla language as a sort of reconciliation tool. And that’s why, when we talk about taking it into the schools—majority of those kids are going to be non-Aboriginal. So, we’re only going to get a small handful that are Aboriginal, and an even smaller handful that are Barngarla. So, when we talk about wanting to expose the school kids to that, I think it does go towards reconciliation, because they’re going to start from a young age, learning about us and our language and our culture and what we’re trying to do. And, yeah, I think that would impact on the whole community.”

#### 3.2.6. Connection to Mind and Emotions

In the context of emotional impacts, several participants described the impacts of language upon mood and affect, noting that people are happier, more open, excited, and more upbeat as a consequence of reclaiming language.

“Everyone’s a bit more pepped up these days, you know. After going to that many meetings and all that and being told where you’re from, everything’s starting to go in the right direction. Language is coming back.”

“You see them when they come back with—got a recharge, got a bit of spark, like a bit of a twinkle in the eye and a sort of upbeat lift. After learning a few more words and all that there, it feels like they come back real pumped, excited. So, to me, when you get excited about something you’re doing, it’s doing good for you.”

“You’re looking at us and seeing us happy, and it’s because we’re genuinely happy. You know, when we’re learning our language and talking it. It is coming from a place where it’s making you happy.”

Participants also spoke about how learning Barngarla language could provide a means for better communicating about emotions such as hopes, dreams, healing, and love.

“At present we still don’t speak our own language. It’s difficult to be able to just open up. I would love to be able to speak to my family in my language and let them know how I feel about them. But I don’t want to talk like that in English, I’d rather talk in my language and let them know how much I love them.”

Perceptions of increasing strength, resilience, personal growth, empowerment, and mending of community also were described as consequences of language reclamation.

“Because it not only allowed us to empower and teach our young fellas, but it got to the community that it wasn’t us butting heads. It’s about the next generation being who they are, and where they’re from, and embracing their heritage, their culture, and using their language.”

Furthermore, involvement with language reclamation improved participants’ attitude and motivation beyond simply their language learning goals.

“Probably the most important thing that I’ve learnt—health-wise—is probably looking after myself more, probably greeting people with a different attitude. Trying to be positive, you know. So, language has done a lot for me although I still don’t know how to speak it.”

“I do look forward to when the language groups are on. Because I can get out and mix with people and, not just with my family group but meeting new people that come. And it just opens you up to be able to share what you have inside you and what you hope, and your thoughts – and it takes you out of your comfort zone too. So that’s the way it’s sort of helped me. I just get up and it’s motivating.”

#### 3.2.7. Connection to Self

Participants expressed how language reclamation impacted upon them personally, making particular reference to identity and cultural pride. For example, participants described how language reclamation had impacted on their wellbeing by strengthening or reinforcing their sense of identity.

“Language plays probably the most important part I believe, in me being who I am and my family being who they are. […] Not [just] learning it but how powerful it is to make you who you are.”

The fundamental importance of language as an aspect of identity, as well as its importance for younger people to build their identity and feelings of pride as Barngarla people was also discussed. As one participant commented, “I think Barngarla language has given people a better sense of identity, where they can feel proud of themselves and who they are and where they come from.” Participants also described increasing cultural pride as a result of being involved in language reclamation activities.

“For me to actually learn about my language and my culture, and my heritage and everything, I reckon it’s just given me the courage; gave me more courage and gave me pride to look at me and think, “I know who I am.” I know my language. Even if I’m not fluent, I can still learn it. And I know just, who I am, and who my ancestors are, and where our boundaries are, and my culture. And I’d love to learn more about it. And I just feel real proud to be who I am.”

“I don’t know how to explain it. It’s like a joyful feeling. Because I’m sharing the language […] And me speaking the language, like my ancestors before me, it’s like a step closer to—I don’t know how to say it. It’s like an overwhelming feeling of happiness and pride in being who I am, and being a Barngarla descendent.”

These seven themes demonstrate a range of ways in which Indigenous language revival can enhance Aboriginal and Torres Strait Islander people’s experiences of wellbeing. 

## 4. Discussion

Language revival is a journey. The advantages of a language revival movement, from the point of view of Indigenous empowerment and wellbeing, go far beyond the actual native speech results, with the process, or the journey, being as important as the goals. This journey is simultaneously for the individual and for the collective or the linguistic community, with potentially different but complimentary effects on individual and communal identity. The main themes emerging from interviews with Barngarla people about the wellbeing impacts of language reclamation revolved around experiences and expressions of connectedness—in particular, connection to spirit and ancestors, Country, culture, family and kinship, community, mind and emotions, and self. Many of these themes are consistent with the findings of the Second National Indigenous Languages Survey (NILS), which identified that 98% of respondents agreed (and 76% strongly agreed) that traditional language use can improve the wellbeing of Aboriginal and Torres Strait Islander peoples (Marmion et al., 2014). Specifically, when asked to describe why language use improves wellbeing, respondents identified two key themes: belonging and empowerment. Most NILS respondents (57%) commented that the use of traditional languages improved people’s sense of belonging to “tradition, culture, ancestor, spirit, family, community, land, and/or country” [3]. This resonates with the findings from our interviews. NILS also reported that 91% of respondents agreed language use was a strong part of their identity and 38% believed that language use empowers people by strengthening their self-esteem, pride and feelings of positivity [3]. 

### 4.1. Aboriginal Social and Emotional Wellbeing

In the context of Aboriginal health, the concept of “social and emotional wellbeing” may be defined as a “multidimensional concept of health that includes mental health, but which also encompasses domains of health and wellbeing such as connection to land or ’country’, culture, spirituality, ancestry, family and community” [36]. This is consistent with the National Aboriginal Community Controlled Health Organisation’s [38] definition of health:

“Aboriginal health means not just the physical well-being of an individual but refers to the social, emotional and cultural well-being of the whole Community in which each individual is able to achieve their full potential as a human being thereby bringing about the total well-being of their Community. It is a whole of life view and includes the cyclical concept of life-death-life.”

While framing wellbeing within Aboriginal world views and culture, it is important to recognise the social, cultural, historical, and political determinants of wellbeing [36]. Social determinants include things such as socioeconomic status, racial discrimination, exposure to trauma and stressful life events, access to community resources, and so forth, which impact upon wellbeing in ways that are concurrent and cumulative. Historical determinants refer to the effects of colonisation and government policies, such as the forced removal of Aboriginal children from their families and communities that resulted in substantial physical displacement and cultural disruption. The context of forced removal is critical to understanding why and how Barngarla communities’ lost fluency in their language, as well as why reviving this language was deemed so important by the Barngarla community members that were interviewed. 

### 4.2. Intergenerational Transfer of Knowledge

Within a context of interrupted family and culture, reviving language has been shown in our study to be a way for Barngarla people to connect across generations. This is illustrated both in the ways that participants reflected on language as a way of reconnecting with heritage and ancestral culture, and in their feelings of obligation in relation to reviving language for Barngarla children and future generations. Participants described children’s interest in language learning as a “thirst” and saw hope in the strength that can come from children and young people driving the desire to learn. The use of song and rhyme was described as a way of embedding language into memory and ensuring that those who are children now might transfer Barngarla language to their children as well.

The context of forced separation from family also highlights the importance that participants placed on “gathering”. In particular, the involvement of all age groups, “the grandmothers…mothers…sons, niece, grandchildren, all mixing,” allowed the sharing, not only of language learning, but of dreams and aspirations for the community into the future. It brought a sense of belonging, of no longer being scattered, and of achieving “something together instead of separately”. Gathering as family and community also provided a way for people to remind one another of their resilience and ongoing connection to a strong family, community, lineage and Country. The feelings of belonging that were fostered through the language-based gatherings allowed participants to “help the group…and the generations to come”.

### 4.3. Language Reclamation and Wellbeing

Throughout the interviews, participants spoke of the inseparable yet multidimensional ways in which language comes to impact upon people’s wellbeing. The frequency with which participants expressed a feeling that “something was missing” from their lives until they began to engage with their language and culture and the ways in which they articulated healing and wholeness in the context of learning language was striking. These included phrasing such as one’s “other half coming in” and language as “the missing part of the jigsaw puzzle”. These findings resonate with those of Anderson, who noted in the context of revitalising Wiradjuri language that, “Learning the language that belongs inside you will heal you. Learning your native language will make you feel more complete” [39].

Our findings indicate that identity and feelings of pride are closely linked with wellbeing. Indeed, Barngarla language was described as the most important part of identity, the power of language to make a person who they are. Furthermore, “the power of who you are and where you come from” was described as being instrumental in bringing people back from depression or despair. There were also participants who overtly linked the “sense of who you are” with “a sense of wellbeing”. Still others described “feelings of happiness and pride in being who I am and being Barngarla descendent”, which illustrates how cultural pride influences wellbeing.

The relationships between wellbeing and connections to Country are also supported by our findings, with “land and language [being] one and the same”. In large part, Barngarla language was experienced as a force that reawakens Barngarla Country at a spiritual level by “breathing language into the land”. In turn, people’s wellbeing was enhanced, as “being in my land and surrounded by my family and learning my language has made me a lot happier”.

For the purposes of the overall study, which aims to measure the impacts of language reclamation on wellbeing, these findings form the basis for identifying “domains of impact” for a psychometric assessment tool. While this process and its findings are presented elsewhere (manuscript forthcoming), the domains of impact that align with our findings include: happiness and excitement; recognition; resilience; optimism and positivity; motivation; empowerment and self-esteem; self-confidence and personal growth; and pride.

### 4.4. Community Impacts of Language Reclamation

In a broader sense, our findings suggest that language reclamation can create flow-on effects at a community level. Participants in our study reflected on how language activities within the community both had shifted negativity and acted as a “reconciliation tool”. This latter trend resonates with Anderson’s findings that the, “introduction of Wiradjuri language, culture and local heritage…raised awareness and pride to the point where racism was significantly reduced in the schools” [39].

Innovative solutions are essential to overcoming the root causes of Aboriginal health inequalities [40]. The empowerment and inclusion of Aboriginal and Torres Strait Islander peoples remains an absolute requirement for developing long-term and sustainable solutions to the precursors of inequity. Increasingly, Indigenous policy is shifting from deficit-based approaches to embrace holistic views of health, as well as outlining the factors which enable and support wellness in the face of an historically hostile and imposed culture. Unique protective factors contained within Indigenous cultures and communities have been sources of strength and healing when the effects of colonisation and oppressive legislation have resulted in loss, grief, and trauma [41]. Language remains one of these foundational protective factors which may provide novel solutions to support communities to grow, survive, and thrive.

### 4.5. Research Benefit

The benefits of this work are of direct relevance to Aboriginal and Torres Strait Islander communities seeking to overcome the negative impact of language loss and social and emotional issues facing them in contemporary Australia. More specifically, the empowerment of Barngarla people, co-occurring alongside their language reclamation, is further contributing to people’s sense of identity, pride and self-esteem. The process of language revival and increasing language use across Eyre Peninsula may enhance the communication and transmission of cultural practice and knowledge across generations. We believe that this project will inform and direct policies that target the preservation, protection and promotion of the rights and freedom of Aboriginal and Torres Strait Islander peoples to use, develop, celebrate, and sustain traditional culture, language, and ways of life.

Engaging Aboriginal community members to conduct as well as participate in Aboriginal health research is both necessary and essential to ensuring that studies are relevant to communities as well as culturally appropriate to the local context. In addition, the active involvement of community members in guiding, shaping, and evaluating research processes helps to build community research capacity more broadly, which in turn allows data to be collected appropriately and analysed and interpreted more accurately. As Mentha and colleagues [34] comment within the context of the Kanyini Qualitative Study: “As with many of our community members, we were worried about research being *done to* Indigenous people without their consent or apparent benefit to them. We were also concerned about Indigenous people being employed as the token ‘blackfellas’ and the potential for community experiences to be interpreted by ‘whitefellas’ who did not really understand our lives” (emphasis in original) [34]. In light of this, the predominantly Aboriginal composition of the Barngarla Language and Wellbeing Study team demonstrates one of the ways in which this study aims to redress the power imbalances that have characterised Aboriginal health research in the past.

### 4.6. Strengths and Limitations

This study is unique in that it documents Aboriginal people’s perceptions of the potential mental health and social and emotional wellbeing impacts of reclaiming their language, following several years of pilot language reclamation activities with a linguist. It further extends the literature by providing an example of how the expertise of Aboriginal community members can form the basis for subsequently developing a quantitative psychometric assessment tool to measure these impacts. By drawing on community members’ experiential expertise (i.e., inviting those who had been involved in pilot language activities to reflect on their experiences and speculate on potential impacts) and thereby centring the voices of Aboriginal community members within the methods development phase of the broader study, it is likely that recruitment into and participation within subsequent aspects of the study also will be enhanced. This study’s commitment to ongoing community engagement with the research processes—including regular community feedback sessions—is another way in which the study aims to ensure genuine research partnerships with Aboriginal communities.

This paper also contributes to Revivalistics [4], a trans-disciplinary field of enquiry surrounding language reclamation (of a no-longer spoken language such as Hebrew or Barngarla language), revitalisation (of a severely endangered language such as Adnyamathanha) and reinvigoration (of an endangered language that still has a high percentage of children speaking it; for example, Welsh and Irish). 

Limitations of the study include the relatively small sample size, and the inclusion of members of only two of the three main Barngarla communities on Eyre Peninsula. Our intention during the course of the study is to encourage other community members to join the project team and study. This would likely include further qualitative interviews to provide opportunities for community members to reflect upon similarities and differences between communities, as well as to continue guiding and evaluating the research and its various processes.

## 5. Conclusions

Language is central to culture, and its loss is a source of grief for many. The main purpose of the Barngarla Language and Wellbeing Study is to assess the effectiveness of language revival in improving the mental health and social and emotional wellbeing of Aboriginal people across Eyre Peninsula. This is a unique project that brings together two distinct, but clearly complementary, scientific paradigms—language revival and population mental health research—which has not previously been attempted. Key outcomes of the study as a whole will include: the first formal test of a causal relationship between language revival and mental health; establishing community-based methods for evaluating mental health interventions; and an innovative wellbeing intervention in high-risk communities. We contend that this will also provide a model for language revival for use in other Indigenous communities. If the hypothesis is supported, we will have gained a powerful, novel tool in improving mental health in high risk communities. Importantly, and as this paper describes, Aboriginal community voices form the central foundation from which the methods for this study are being developed. The study’s commitment to ongoing community input to guide and shape the research processes aims to ensure community relevance and cultural appropriateness at all stages of the study.
